# Stability Properties of Geometrothermodynamic Cosmological Models

**DOI:** 10.3390/e25101391

**Published:** 2023-09-28

**Authors:** Nurzada Beissen, Medeu Abishev, Manas Khassanov, Temirbolat Aitassov, Sagira Mamatova, Saken Toktarbay

**Affiliations:** 1 Institute for Experimental and Theoretical Physics, Al-Farabi Kazakh National University, Almaty 050040, Kazakhstan; nurzada.beissen@kaznu.edu.kz (N.B.); medeu.abishev@kaznu.kz (M.A.); khassanov.manas@kaznu.kz (M.K.); mamatova_sagira@live.kaznu.kz (S.M.); 2 Institute of Nuclear Physics, 1 Ibragimova St., Almaty 050032, Kazakhstan; 3 Department of Mathematics, Physics and Informatics Teaching Methods, Abai Kazakh National Pedagogical University, Almaty 050010, Kazakhstan; temirbolat.aytasov@physics.kz; 4 Department of Physics, Kazakh National Women’s Teacher Training University, Almaty 050000, Kazakhstan

**Keywords:** cosmology, geometrothermodynamics

## Abstract

We consider a particular isotropic and homogeneous cosmological model, in which the equation of state is obtained from a thermodynamic fundamental equation by using the formalism of geometrothermodynamics (GTD). The model depends effectively on three arbitrary constants, which can be fixed to reproduce the main aspects of the inflationary era and the ΛCDM paradigm. We use GTD to analyze the geometric properties of the corresponding equilibrium space and to derive the stability properties and phase transition structure of the cosmological model.

## 1. Introduction

One of the most interesting results of relativistic cosmology in the framework of Einstein’s gravity theory is the standard cosmological model. It is based on the cosmological principle, stating that at large scales, the Universe is homogeneous and isotropic. Moreover, to implement Einstein’s equations, it is necessary to assume a model for the large-scale structure of the universe. The simplest choice is that of an energy–momentum tensor corresponding to a perfect fluid. The resulting field equations are known as the Friedmann equations, which relate the density and pressure of the perfect fluid with the expansion rate of the universe. Apart from the Friedmann equations, it is necessary to add an equation of state in order to construct the details of the ΛCDM scenario, which is considered the best model to explain the evolution of the universe [1].

As an alternative way to construct cosmological models, it has been proposed to use a fundamental equation, so that the entire universe can be considered as a thermodynamic system. The fundamental equation, in turn, is obtained by using the theory of GTD [2,3,4,5].

Indeed, the idea of GTD consists in analyzing the thermodynamic properties of a system by using concepts of contact geometry and Riemannian geometry. To this end, it is necessary to introduce the concept of equilibrium space, an *n*-dimensional space whose points correspond to the states of equilibrium of the system, and the 2n+1-dimensional phase space, where the thermodynamic potential, extensive variables, and intensive variables are considered as independent coordinates. At the level of the equilibrium space only, it is possible to introduce the formalism of thermodynamic geometry, which consists in introducing metrics in such a way that the equilibrium space becomes a Riemannian manifold. The first Riemannian structure of thermodynamic geometry was proposed by Rao [6] in 1945 by identifying the components of the Fisher information matrix as the components of a Riemannian metric, which is currently known as the Fisher–Rao metric. In 1975, Weinhold introduced a different metric, whose components are the Hessian of the internal energy [7,8,9,10]. Then, in 1979, Ruppeiner proposed a different metric with components given as the Hessian of the entropy [11,12]. It turned out that the Ruppeiner metric is the thermodynamic limit of the statistical Fisher–Rao metric (see [13] for a review).

The approach of geometrothermodynamics is different and was proposed by Quevedo in 2007 by using the additional structure of the phase space and assuming as fundamental principle the Legendre invariance of the metrics of the phase space [14]. As a result, the GTD metrics determine Riemannian structures, which are completely different from those used in thermodynamic geometry.

We present in this work an introduction to the theory of GTD by considering the exact mathematical definitions of the phase space and the equilibrium space. GTD is based on the physical invariance of classical thermodynamics with respect to Legendre transformations, i.e., with respect to the choice of thermodynamic potential.

This work is organized as follows. In Section 2, we review the standard cosmological model. We emphasize the fact that an equation of state is necessary in order to integrate the corresponding Friedmann equations. In Section 3, we review the main aspects of the geometric structure of GTD. We then explain, in Section 4, how to construct geometrothermodynamic cosmological models and consider a particular example that contains the ΛCDM model as particular case and the inflationary era as another particular case. In Section 5, we focus on the study of the stability properties of the model by using the geometric structure of the GTD theory. Finally, in Section 6, we summarize our results.

## 2. The Standard Cosmological Model

According to observations, at scales on the order of hundreds of megaparsecs, the universe satisfies the cosmological principle. This means that the spacetime of the universe can be split into hypersurfaces of constant time, which are isotropic and homogeneous. This is then taken into relativistic cosmology as an assumption for the entire universe and its complete evolution. In turn, this assumption fixes the form of the line element of the spacetime that can be written as [1,15,16]

(1) $$ds^{2}=-dt^{2}+R^{2}\left(t\right)\left[\frac{dr^{2}}{1-kr^{2}}+r^{2}\left(d\theta^{2}+\sin^{2}\theta d\phi^{2}\right)\right]\;,$$

which is known as the Friedmann–Lemaître–Robertson–Walker (FLRW) line element. Here, R(t) is the scale factor and *k* is a constant that represents the curvature of the hypersurfaces of constant time.

Furthermore, Einstein’s equations with a perfect fluid as the source of gravity lead to the Friedmann equations (we use units with G=c=1)

(2) $$\frac{{\dot{R}}^{2}}{R^{2}}+\frac{k}{R^{2}}=\frac{8\pi }{3}\rho \;,$$

(3) $$\frac{\ddot{R}}{R}=-\frac{4\pi }{3}\left(\rho +3p\right)\;,$$

where ρ is the density and *p* the pressure of the fluid. Here, a dot represents derivation with respect to the time parameter *t*.

The following step to develop the cosmological model consists in integrating Friedmann equations. To this end, it is necessary to postulate an additional equation, which is usually an equation of state that relates density and pressure. The simplest choice is the barotropic equation of state p=wρ, where *w* is the constant barotropic factor. It then turns out that Friedman equations can be integrated for different values of the barotropic factor *w*. According to observations, the standard cosmological model highlights three different epochs for the evolution of the universe, namely the epoch of radiation (w=1/3), the matter-dominated era (w=0), and the dark-energy-dominated epoch (w=−1).

Geometrothermodynamics proposes an alternative approach to integrate Friedmann’s equations. The idea is to replace the equation of state p=wρ by a fundamental equation that describes a thermodynamic system. The difference is that with a fundamental equation, it is also possible to derive other properties of the system, such as additional thermodynamic variables, response functions, phase transitions, etc. In turn, the fundamental equation can be obtained from the GTD theory by applying a variational principle.

## 3. The Theory of GTD

There are several proposals to apply Riemannian differential geometry in classical thermodynamics. One of the most popular proposals consists in introducing Riemannian metrics into the equilibrium space, whose points represent equilibrium states of the corresponding thermodynamic system. In particular, Hessian metrics have also been used extensively in statistical physics and information theory [7,8,9,10,11,12,13,17,18,19,20].

The theory of GTD [14] uses concepts of contact geometry to guarantee that the formalism itself satisfies the property of ordinary thermodynamics of being invariant under the action of Legendre transformations. Therefore, GTD is the only theory that takes into account this property, which from a physical point of view means that the theory does not depend on the choice of thermodynamic system [21]. This is an essential symmetry of classical thermodynamics that makes it possible to treat GTD as a theory from the mathematical point of view in a way that resembles the mathematical construction of field theories.

The starting point of the geometric construction of GTD is the phase space that is defined as follows. The manifold (P,Θ,G) is called a Riemannian contact manifold if P is a 2n+1-dimensional differential manifold, Θ is a contact 1-form satisfying the condition $\Theta \wedge {\left(d\Theta \right)}^{n}=\Theta \wedge d\Theta \wedge d\Theta ...\neq 0$, and *G* is a Riemannian metric on P. The interesting point of this construction is that it is always possible to introduce coordinates $Z^{A}=\left(\Phi ,E^{a},I^{a}\right)$, with *a* ranging from 1 to *n*, such that the contact 1-form can be written as $\Theta =d\Phi -\delta_{ab}I^{a}dE^{b}$ with $\delta_{ab}=\mathrm{diag}\left(1,\ldots ,1\right)$. These coordinates are called canonical Darboux coordinates. In these coordinates, the condition $\Theta \wedge {\left(d\Theta \right)}^{n}\neq 0$ is equivalent to $d\Phi \wedge dE^{1}\wedge ...\wedge dE^{n}\wedge dI^{1}\wedge ...\wedge dI^{n}\neq 0$, meaning that the volume element of P is nonzero.

We say that the Riemannian contact manifold P,Θ,G) is a thermodynamic phase space if it is invariant under Legendre transformations of the form [14]

(4) $$Z^{A}\to {\tilde{Z}}^{A}=\left(\tilde{\Phi },{\tilde{E}}^{a},{\tilde{I}}^{a}\right)$$

with

(5) $$\Phi =\tilde{\Phi }-\delta_{kl}{\tilde{E}}^{k}{\tilde{I}}^{l}\;,\;E^{i}=-{\tilde{I}}^{i},\;\;E^{j}={\tilde{E}}^{j},\;I^{i}={\tilde{E}}^{i},\;\;I^{j}={\tilde{I}}^{j}\;,$$

where k,l=1,…,i and i∪j is any disjoint decomposition of the set of indices {1,…,n}.

Interestingly, the contact 1-form Θ is form-invariant with respect to the above Legendre transformations, in the sense that under a Legendre transformation $Z^{A}\to {\tilde{Z}}^{A}$, it behaves as $\Theta \to \tilde{\Theta }=d\tilde{\Phi }-\delta_{ab}{\tilde{I}}^{a}d{\tilde{E}}^{b}$. However, the components of the metric $G_{AB}$ are not form invariant, in general. Nevertheless, it is possible to find the conditions for $G_{AB}$ to be Legendre-invariant, which can be solved and yield a set of three different metrics, whose line elements can be represented as

(6) $$G^{{}^{I}}={\left(d\Phi -I_{a}dE^{a}\right)}^{2}+\left(\beta_{ab}E^{a}I^{b}\right)\left(\delta_{cd}dE^{c}dI^{d}\right)\;,$$

(7) $$G^{{}^{II}}={\left(d\Phi -I_{a}dE^{a}\right)}^{2}+\left(\beta_{ab}E^{a}I^{b}\right)\left(\eta_{cd}dE^{c}dI^{d}\right)\;,$$

(8) $$G^{III}={\left(d\Phi -I_{a}dE^{a}\right)}^{2}+\sum_{a=1}^{n}\beta_{a}\left(E_{a}I_{a}\right)dE^{a}dI^{a}\;,$$

where $\eta_{ab}=\mathrm{diag}\left(-1,1,\cdots ,1\right)$, $\beta_{ab}=\mathrm{diag}\left(\beta_{1},\ldots ,\beta_{n}\right)$, and $\beta_{a}$ are the coefficients of quasi-homogeneity of the variables $E^{a}$ within the fundamental equation $\Phi =\Phi (E^{a})$ [22].

The quasi-homogeneity coefficients are related to the symmetry properties of the fundamental equation, which is demanded to be a quasi-homogeneous function of degree $\beta_{\Phi }$, i.e., under the rescaling $E^{a}\to \lambda^{\beta_{a}}E^{a}$, it behaves as

(9) $$\Phi \left(\lambda^{\beta_{a}}E^{a}\right)=\lambda^{\beta_{\Phi }}\Phi \left(E^{a}\right),$$

where λ is a positive real constant and $\beta_{a}$ positive or negative real constants.

The second main component of GTD is the equilibrium space, which is a Riemannian submanifold of the phase space (E,g)⊂(P,Θ,G), defined by the conditions

1. φ:E⟶P, i.e., $\varphi :\left\{E^{a}\right\}⟼\left\{\Phi \left(E^{a}\right),E^{a},I^{a}\left(E^{a}\right)\right\}$ is a smooth embedding map.
2. $\varphi^{*}\left(\Theta \right)=0$, i.e., $d\Theta =I_{a}dE^{a}$ on E, which is equivalent to saying that on E, the first law of thermodynamics is satisfied, where $E^{a}$ represent the extensive thermodynamic variables and $I_{a}=\frac{\partial \Phi }{\partial E^{a}}$ are the corresponding dual variables.
3. The pullback $\varphi^{*}$ induces metrics of E by means of $g=\varphi^{*}\left(G\right)$, i.e.,
  (10) $$g_{ab}=\frac{\partial Z^{A}}{\partial E^{a}}\frac{\partial Z^{B}}{\partial E^{b}}G_{AB}=Z_{,a}^{A}Z_{,b}^{B}G_{AB}\;.$$
  In particular, the metrics generated by the pullback of (6)–(8) are
  (11) $$g_{ab}^{I}=\beta_{\Phi }\Phi \delta_{a}^{\;c}\frac{\partial^{2}\Phi }{\partial E^{b}\partial E^{c}},$$
  (12) $$g_{ab}^{II}=\beta_{\Phi }\Phi \eta_{a}^{\;c}\frac{\partial^{2}\Phi }{\partial E^{b}\partial E^{c}},$$
  (13) $$g^{III}=\sum_{a=1}^{n}\beta_{a}\left(\delta_{ad}E^{d}\frac{\partial \Phi }{\partial E^{a}}\right)\delta^{ab}\frac{\partial^{2}\Phi }{\partial E^{b}\partial E^{c}}dE^{a}dE^{c}\;,$$
  respectively, where $\delta_{a}^{\;c}=\mathrm{diag}\left(1,\cdots ,1\right)$, $\eta_{a}^{\;c}=\mathrm{diag}\left(-1,1,\cdots ,1\right)$, and $\beta_{\Phi }$ is the quasi-homogeneity index of the function $\Phi (E^{a})$.
4. φ is a harmonic map, i.e., the action $I_{g}=\int_{E}d^{n}E\sqrt{\left|\det(g_{ab})\right|}$ is stationary, i.e.,
  (14) $$\frac{\delta I_{g}}{\delta Z^{A}}=\frac{1}{\sqrt{\left|\det(g_{ab})\right|}}{\left(\sqrt{\left|\det(g_{ab})\right|}\;\;g^{ab}Z_{,a}^{A}\right)}_{,b}+\Gamma_{\;BC}^{A}Z_{,b}^{B}Z_{,c}^{C}g^{bc}=0\;,$$
  where
  (15) $$\Gamma_{\;bc}^{a}=\frac{1}{2}G^{ad}\left(G_{db,c}+G_{dc,b}-G_{bc,d}\right)$$
  are the Christoffel symbols of the metric $G_{ab}$ of the phase space.

The solution to this equation $Z^{A}=Z^{A}\left(E^{a}\right)$ includes the function $\Phi =\Phi (E^{a})$, which could be interpreted as a fundamental equation if it satisfies the laws of thermodynamics. A particular solution for n=2 is

(16) $$\Phi =c_{1}\ln\left(E^{1}+\frac{\alpha }{E^{2}}\right)+c_{2}\ln\left(E^{2}-\beta \right)\;,$$

where $c_{1}$, $c_{2}$, α, and β are constants.

Although this solution is valid only in the case n=2, it is possible to generalize it to include higher dimensions as follows

(17) $$\Phi =c_{1}\ln\left(E^{1}+\frac{\alpha }{E^{l}}\right)+\sum_{k=2}^{n}c_{k}\ln\left(E^{k}-\beta_{k}\right)\;,$$

where l∈(1,2,…,n). In the following section, we see that the case with n=2 can be applied in the context of relativistic cosmology with two effective fluids. In general, however, we believe that the solution for higher dimensions could be used in case additional effective fluids are necessary.

## 4. A Cosmological Model Based on GTD

As mentioned above, the idea of using GTD to construct cosmological models consists in endowing the Friedmann equations with a fundamental equation derived from GTD. Using the solution (16) with Φ=S, $E^{1}=U$, and $E^{2}=V$, the GTD cosmological model is based on the equations

(18) $$\frac{{\dot{R}}^{2}}{R^{2}}+\frac{k}{R^{2}}=\frac{8\pi }{3}\rho \;,$$

(19) $$\frac{\ddot{R}}{R}=-\frac{4\pi }{3}\left(\rho +3p\right)\;,$$

(20) $$S=c_{1}\ln\left(U+\frac{\alpha }{V}\right)+c_{2}\ln\left(V-\beta \right)\;,$$

where we interpret *S* as the entropy of the universe, U=ρV its internal energy, and $V=V_{0}R^{3}\left(t\right)$ its volume. It has been shown in [23] that the above system can be used to construct an inflationary model and to reproduce the standard ΛCDM model. In fact, since the fundamental Equation (20) is assumed to satisfy the first law of thermodynamics,

(21) $$dS=\frac{1}{T}dU+\frac{p}{T}dV\;,$$

we can compute the thermodynamic temperature

(22) $$T=\frac{U}{c_{1}}+\frac{\alpha }{c_{1}V}\;,$$

and the pressure

(23) $$p=\frac{c_{2}UV^{2}+\alpha \left[\beta c_{1}+\left(c_{2}-c_{1}\right)V\right]}{c_{1}V^{2}\left(V-\beta \right)}\;.$$

In the particular case with α=β=0, we obtain

(24) $$U=c_{1}T\;,\;pV=\frac{c_{2}}{c_{1}}U\;,$$

which implies that $p=\frac{c_{2}}{c_{1}}\rho$. This is a barotropic equation of state, with $w=\frac{c_{2}}{c_{1}}$ as the barotropic factor. Consequently, this model is equivalent to the standard cosmological model, reproducing the evolution epochs for different values of the ration $\frac{c_{2}}{c_{1}}$.

In the general case with α≠0 and β≠0, it has been shown that one can obtain an effective inflationary model, which is in accordance with the standard inflationary parameter [24,25,26], namely, the beginning and end of inflation is limited by the values $t_{i}∼10^{-36}$ s and $t_{f}∼10^{-32}$ s, a period during which the universe expanded exponentially from $R(t_{i})$ to $R(t_{f})$ according to the relationship $R\left(t_{f}\right)=e^{60}R\left(t_{i}\right)$. These physical values are part of the model under the assumption that the free parameters are chosen as

(25) $$\frac{c_{2}}{c_{1}}=-\frac{8}{9}\;,\;\alpha ≃10^{-78}\;{\mathrm{Jm}}^{3}\;,\;\beta ≃10^{-84}\;m^{3}\;.$$

The parameter α is interpreted as related to the interaction of the particles that conform the cosmic fluid. The parameter $\beta =10^{-84}\;m^{3}$ is related to the volume of the universe at the beginning of inflation. The values of these parameters represents a prediction of the model.

## 5. Stability Properties of the Model

In GTD, the stability properties a thermodynamic system can be determined by analyzing the properties of the curvature of the equilibrium space. In the last section, we used the fundamental Equation (20) to construct a cosmological model, in which the entire universe is the thermodynamic system with two degrees of freedom, n=2. Accordingly, we have to investigate the curvature properties of the metrics (11)–(13). Using the notations Φ=S, $E^{1}=U$, and $E^{2}=V$, we obtain

(26) $$\begin{array}{ccc} g^{I} & = & \beta_{S}S\left[S_{,UU}{\left(dU\right)}^{2}+2S_{,UV}dUdV+S_{,VV}{\left(dV\right)}^{2}\right]\;, \end{array}$$

(27) $$\begin{array}{ccc} g^{II} & = & \beta_{S}S\left[-S_{,UU}{\left(dU\right)}^{2}+S_{,VV}{\left(dV\right)}^{2}\right]\;, \end{array}$$

(28) $$\begin{array}{ccc} g^{III} & = & \beta_{U}US_{,U}S_{,UU}{\left(dU\right)}^{2}+\beta_{V}VS_{,V}S_{,VV}{\left(dV\right)}^{2}\\ & & +\left[\beta_{U}\left(US_{,U}\right)+\beta_{V}\left(VS_{,V}\right)\right]S_{,UV}dUdV\;, \end{array}$$

where $S_{,U}=\frac{\partial S}{\partial U}$, etc.

The components of the first metric $g^{I}$ can be written as $g_{ab}^{I}=\beta_{S}S\frac{\partial^{2}S}{\partial E^{a}\partial E^{b}}$, meaning that it is conformal to a Hessian metric with the entropy as the Hessian potential. In this sense, the metric $g^{I}$ is conformal to the Ruppeiner metric, which is not invariant under Legendre transformations. We see that the role of the conformal factor *S* consists in guaranteeing the Legendre invariance of the GTD metric $g^{I}$. This analogy between the line element $g^{I}$ and the Ruppeiner line element is the reason why in most cases the results obtained from $g^{I}$ coincide with those obtained in thermodynamic geometry.

The line element $g^{II}$ also contains the entropy as the conformal factor. However, the metric itself is not Hessian due to the presence of the pseudo-Euclidean metric $\eta_{a}^{\;c}$ in the original Formula (12). This means that there is no analogue metric in thermodynamic geometry. Interestingly, the determinant of the metric contains the term $S_{,UU}S_{,VV}$, which appears then in the denominator of the scalar curvature $R^{II}$ (see below) and partially determines the phase transitions of the corresponding system. In concrete examples, one can see that the solutions of the equation $S_{,UU}S_{,VV}=0$ correspond to divergences of the response functions, such as the heat capacity, i.e., to phase transitions.

The line element $g^{III}$ represents the most general metric, which is invariant with respect to partial Legendre transformations. It cannot be related in any way to the Hessian metrics of thermodynamic geometry. As shown below, this metric is free of curvature singularities if $S_{UV}\neq 0$, a condition that is essential for the determination of the stability properties of the corresponding system.

The singularity structure of the above metrics is determined from the behavior of the corresponding Ricci scalars. In fact, in two dimensions, there is only one independent component of the curvature tensor, and so all the algebraic curvature scalars should be proportional the Ricci scalar. Using the Euler identity in the form [27]

(29) $$\beta_{U}US_{,U}+\beta_{V}VS_{,V}=\beta_{S}S\;,$$

the computation of the Ricci scalars yields

(30) $$R^{I}=\frac{N^{I}}{2\beta_{S}S^{3}{\left[S_{,UU}S_{,VV}-{\left(S_{,UV}\right)}^{2}\right]}^{2}}\;,$$

(31) $$R^{II}=\frac{N^{II}}{2\beta_{S}S^{3}{\left(S_{,UU}S_{,VV}\right)}^{2}}\;,$$

(32) $$R^{III}=\frac{N^{III}}{{\left[\beta_{S}^{2}S^{2}{\left(S_{,UU}\right)}^{2}-4\beta_{U}\beta_{V}UVS_{,U}S_{,V}S_{,UU}S_{,VV}\right]}^{3}}\;,$$

respectively, where the functions $N^{I}$, $N^{II}$, and $N^{III}$ are functions of *S* and its derivatives.

A detailed analysis of the form of the denominators of the expressions (30)–(32) shows that the singularities are determined by the conditions [28,29]

(33) $$\begin{array}{ccc} & & S_{,UU}S_{,VV}-{\left(S_{,UV}\right)}^{2}=0\;, \end{array}$$

(34) $$\begin{array}{ccc} & & S_{,UU}S_{,VV}=0\;, \end{array}$$

(35) $$\begin{array}{ccc} & & S_{,UV}=0\;, \end{array}$$

respectively, which can be interpreted in terms of the stability conditions and phase transition structure of the corresponding system.

Notice that the above conditions are not independent, because the validity of any two of them implies the validity of the third one. However, for the analysis of concrete examples, it is convenient to consider all three conditions separately.

We now investigate the conditions (33) and (34). First, consider the limiting case α=β=0. Then,

(36) $$S_{,UU}S_{,VV}-{\left(S_{,UV}\right)}^{2}=\frac{c_{1}c_{2}}{U^{2}V^{2}}=0\;,$$

(37) $$S_{UU}=-\frac{c_{1}}{U^{2}}\;,\;S_{,VV}=-\frac{c_{2}}{V^{2}}\;,\;S_{UV}=0\;.$$

We see that in general, for nonzero values of $c_{2}$, the singularity conditions are not satisfied, implying that during the period corresponding to the ΛCDM model, from a thermodynamic point of view, no instabilities and no phase transitions can occur.

The lack of phase transitions means that the model cannot represent the crossing between different eras of the evolution of the Universe. However, one can expect that parametrizations can be used to describe transitions between eras, in a similar way as it is performed in the standard cosmological model.

Now, we consider the general case (α≠0,β≠0), which corresponds to an inflationary model. A straightforward calculation with the fundamental Equation (20) shows that

(38) $$S_{UU}=-\frac{c_{1}V^{2}}{{\left(UV+\alpha \right)}^{2}}\;,\;S_{UV}=\frac{c_{1}\alpha }{{\left(UV+\alpha \right)}^{2}}\;,$$

so that these derivatives do not lead to nontrivial zeros. To express the remaining conditions in a compact form, in the final result, we replace the expression with the pressure (23) and the value $c_{2}=-\frac{8}{9}c_{1}$ we obtained for the inflationary era. Then,

(39) $$S_{,UU}S_{,VV}-{\left(S_{,UV}\right)}^{2}=0⟷pV^{3}-\alpha V+2\alpha \beta =0\;,$$

(40) $$S_{,VV}=0⟷9p^{2}V^{5}+18\alpha \beta pV^{2}-17\alpha^{2}V+18\alpha^{2}\beta =0\;.$$

**Figure 1:**
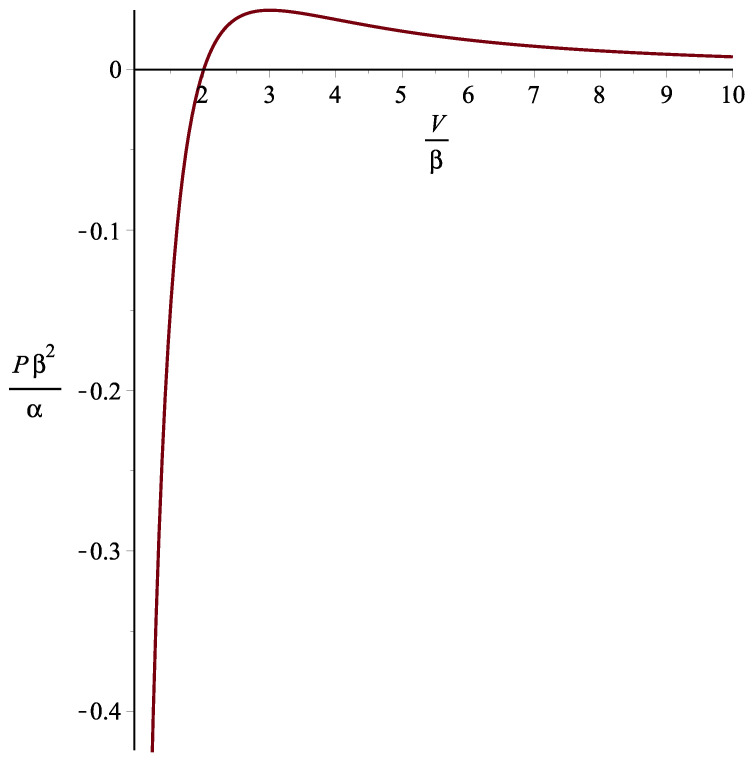
Representation of the condition (39) for the inflationary era. For concreteness, we assume that α is positive.

Condition (39) means that there are values for the pressure at which instabilities can take place. In Figure 1, we show the behavior of the pressure in terms of the volume.

We choose the values of the volume as V>β in order for the fundamental Equation (20) to be well defined. Also, we expect that during inflation, the pressure is negative for the universe to expand. We see that only in the interval V∈(β,2β) is the pressure negative. Since $\beta =10^{-84}\;m^{3}$ was interpreted as the volume of the universe at the beginning of inflation, we conclude that the instabilities can occur only at the onset of inflation. Later on, no instabilities can occur.

Consider now the condition (40). We solve this condition with respect to the pressure and obtain

(41) $$\frac{p_{1,2}}{\alpha }=-\beta \pm \frac{1}{V^{3}}\left[3\beta +\sqrt{9\beta^{2}-18\beta V+17V^{2}}\right]\;.$$

**Figure 2:**
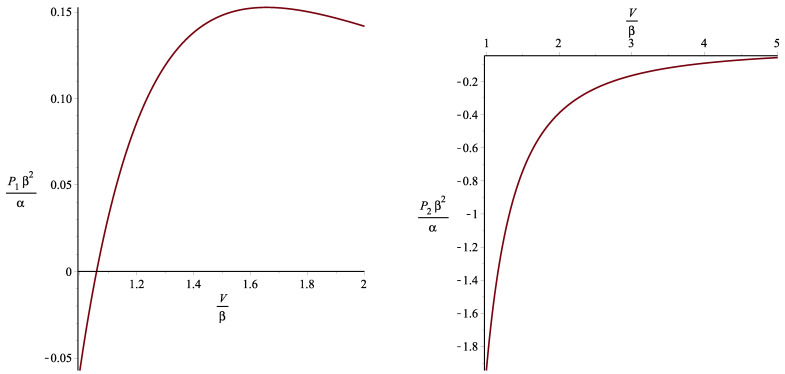
Representation of the condition (40) for the inflationary era. The values of the pressure $p_{1}$ and $p_{2}$ are the solutions of this condition. For concreteness, we assume that α is positive.

In Figure 2, we show the behavior of $p_{1}$ and $p_{2}$ in terms of the volume.

The plot of the solution $p_{1}$ (left panel) shows that the pressure is negative only within the interval $\left(\beta ,\frac{18}{17}\beta \right)$, i.e., in the vicinity of the onset of inflation. This means that a phase transition can take place at the moment where the inflation starts. The solution $p_{2}$ (right panel) is always negative. This means that such a phase transition can occur at any time during the inflation.

In conclusion, the analysis of the Ricci scalars of the metrics of the equilibrium space shows that there can be three different classes of critical points, corresponding to the different singularity conditions, at which instabilities and phase transitions can take place. Two of these critical points occur in the vicinity of the inflation onset, i.e., they could be interpreted as describing the thermodynamic beginning of inflation. The third critical point can occur at any time during inflation and would indicate the occurrence of a phase transition.

The phase transitions that occur before and during inflation cannot be described within the frame of the GTD theory, because phase transitions lead to the appearance of non-equilibrium states. To investigate such states. it would be necessary to generalize the geometric structure of GTD in such a way that non-equilibrium states are points of a deformed equilibrium space. This task, however, is well beyond the scope of the present work.

## 6. Conclusions

This work is dedicated to the study of geometrothermodynamic cosmology. First, we present the essentials of the standard cosmological model, emphasizing the fact that an additional equation is necessary in order to be able to integrate Friedmann equations. In the case of the standard ΛCDM scenario, the additional equation is a barotropic equation of state. In the case of geometrothermodynamic cosmology, it is a fundamental equation, which is a solution of a set of differential equations derived from the theory of GTD.

We reviewed the geometric structure of GTD, including the construction of the phase space, where the conditions of Legendre invariance are implemented as coordinate transformations, and the equilibrium space, where the laws of thermodynamics are valid, and the metrics and geometric properties are derived from the knowledge of the fundamental equation of the system under consideration. We investigated in detail the three different metrics that can be determined from the fundamental equation of a particular cosmological model that includes the inflationary era and the epochs of the standard ΛCDM paradigm.

Furthermore, we analyzed in detail the curvature singularities of the equilibrium space and found that there are three critical points where instabilities and phase transitions can take place. We use as a criterion for determining the physical meaning of the critical points the condition that the pressure be negative. This is in accordance with our physical expectations that expansion is generated effectively by a negative pressure. It turns out that two critical points occur practically at the beginning of inflation; so, we can interpret them as the generators of inflation. The third critical point can be interpreted as a phase transition that can occur at any time during inflation.

From the above results, we see that the application of GTD in cosmology allows us to explore the Universe from a different perspective. We established that the beginning of inflation is a thermodynamic process associated with a phase transition. This opens the possibility of exploring the pre-inflationary era by using non-equilibrium thermodynamics and non-equilibrium GTD. After inflation, the evolution of the Universe corresponds effectively to the thermodynamic evolution of an ideal barotropic gas, with a different barotropic factor for each era of evolution. This is a novel aspect that deserves further investigation.

## Data Availability

Not applicable.
